# Feasibility and Efficacy of a Novel Mindfulness App Used With Matcha Green Tea in Generally Healthy Adults: Randomized Controlled Trial

**DOI:** 10.2196/63078

**Published:** 2024-12-10

**Authors:** Ryohei Tanaka-Kanegae, Koji Yamada, Chad M Cook, Traci M Blonquist, Kristen D Taggart, Koichiro Hamada

**Affiliations:** 1 Saga Nutraceuticals Research Institute Otsuka Pharmaceutical Co Ltd Saga Japan; 2 Otsuka Holdings Co Ltd Tokyo Japan; 3 Biofortis, Inc Addison, IL United States

**Keywords:** mindfulness, guided tea meditation, meditation, matcha, green tea, mobile app, smartphone, stress, mood, mHealth, mobile health, well-being, wellness

## Abstract

**Background:**

Mindfulness practices, such as breathing meditation (BM), reduce stress and enhance mood. One such practice is mindful eating, where a practitioner focuses on the five senses while eating or drinking. A novel set of prototypes has been developed, incorporating principles of mindful eating. These prototypes include matcha green tea and a mobile app that provides audio guidance for meditation during the preparation and consumption of the beverage (hereafter referred to as guided tea meditation [GTM]).

**Objective:**

This study assessed the feasibility and efficacy of GTM, evaluating meditation time, frequency, and prototype acceptability over 8 weeks, alongside changes in stress and mood. Additionally, other benefits of GTM were explored.

**Methods:**

A comparator group was established in which participants performed traditional BM without an app or audio guide (active control). This unblinded randomized controlled trial involved 100 healthy American volunteers (n=49 GTM, n=51 BM). During the 8-week study period, participants were encouraged to perform either GTM or BM for 10 minutes daily. The meditation activity was self-reported the following day. Only the GTM group assessed the prototype acceptability. The Perceived Stress Scale-10 was used to measure stress levels, while the Two-Dimensional Mood Scale was used to evaluate mood changes. Other meditation benefits were explored using a questionnaire. All questionnaires were presented and completed via an app. An intention-to-treat analysis was performed.

**Results:**

No significant between-group differences were found in total meditation time (*P*=.15) or frequency (*P*=.36). However, the weekly time and frequency of the GTM group remained above 50 minutes per week and 4 days per week, respectively. Over half of the GTM participants (≥28/49, ≥57%) accepted the prototype. The GTM group exhibited significant stress reductions at weeks 4 and 8 (both *P*<.001), similar to the BM group. Improvements in mood metrics were observed after a single GTM session on days 1 and 56, similar to the BM group. Moreover, increases in premeditation scores for relaxed and calm from day 1 to day 56 were significantly higher for the GTM group (*P*=.04 and .048, respectively). The majority of participants (≥25/49, ≥51%) assigned to GTM experienced positive changes in happiness, time management, quality of life, relationships, sleep, and work performance as they continued meditating. However, no significant between-group differences were found in these exploratory outcomes (*P*>.08).

**Conclusions:**

We believe that GTM exhibits good feasibility. Meanwhile, GTM reduced stress, improved mood, and let the practitioners feel other benefits, similar to BM. Long-term practitioners of GTM may even feel more relaxed and calmer in the state of premeditation than those who practice BM.

**Trial Registration:**

ClinicalTrials.gov NCT05832645; https://clinicaltrials.gov/study/NCT05832645

## Introduction

### Background

Stress management or reduction is an important skill for many individuals because of the increasing occurrence of stress, anxiety, and depression. Indeed, approximately one-third of the global population experiences stress [1]. The United States is one of the most stressed nations in the world, with more than three-fourths of American adults reporting symptoms of stress, including headache, tiredness, and sleep problems [2]. This problem was exacerbated by the COVID-19 pandemic and people still struggle to adapt to the rapid changes caused by the pandemic [3-5]. Stress not only affects mood and mental health but also physical health, either directly by disturbing the autonomic nervous and neuroendocrine systems or indirectly by causing changes in health behaviors [6]. Thus, it is important to assess and encourage stress-lowering techniques to achieve well-being and tranquility in life.

Mindfulness-based approaches have been increasingly used to help individuals respond more effectively to stress and other negative internal experiences. Participation in an 8-week mindfulness-based stress reduction (MBSR) program lowered stress and anxiety symptoms [7,8]. Breathing meditation (BM; referred to as sitting meditation with awareness of breathing in the MBSR program) is the main technique that program participants are encouraged to practice daily [9]. During the meditation, a practitioner pays attention to bodily sensations that accompany breathing, and whenever they notice their mind wandering, they note what diverts their attention and subsequently redirect it to breathing [10]. Studies have demonstrated the positive effects of a 10-minute BM on emotion regulation and attention control [11,12].

MBSR programs provide a variety of mindfulness techniques other than BM and participants are encouraged to seek as many opportunities as possible to practice these techniques in their daily lives [10]. One such technique is mindful eating, in which a practitioner pays attention to the sight, smell, taste, texture, and temperature of food, as well as their thoughts and feelings while eating [13]. Several studies have shown a correlation between the degree of beneficial traits or effects that practitioners can obtain from meditative training and the total time or frequency of meditation [12,14,15]. Therefore, it seems reasonable to use time spent eating and drinking, which is an essential part of living, as an opportunity to practice mindfulness.

Japan has a long-standing tradition of tea ceremony where matcha green tea is served to guests. The ceremony has been shown to have a calming effect on participants [16,17]. Regarding taste, matcha green tea tastes sweet and umami (savory), as well as bitter [18]. We believe this differentiates matcha green tea from other beverages such as regular green tea and coffee, enabling practitioners to cultivate their awareness while focusing on the different tastes. Hence, we developed a dedicated matcha green tea for mindful eating as a prototype. Moreover, a prototype mobile app that provides audio guidance to perform meditation while making and drinking the beverage was developed. Hereafter, the whole program for stress management provided by the novel set of prototypes is referred to as guided tea meditation (GTM).

There are several commercial mindfulness apps that guide mindful eating. However, to the best of our knowledge, they are mainly intended for weight control [19]. Their efficacy on mental health, such as stress levels and mood, is limited [20], and no commercial products exist that incorporate an app and food for cultivating mindfulness.

### Objective

This study aimed to investigate the feasibility and efficacy of GTM. To assess feasibility, the time and frequency of meditation using the prototypes were evaluated over 8 weeks. Additionally, the acceptability of the prototypes was evaluated. To assess efficacy, changes in stress levels over 8 weeks and changes in mood before and after single and multiple meditation sessions were investigated. Moreover, other benefits of GTM were explored using a questionnaire. The findings of this study may facilitate the collaboration between the two different fields, mobile health and food science.

## Methods

### Ethical Considerations

This study was approved by the Sterling IRB (Atlanta, Georgia; approval: 10829), registered at ClinicalTrials.gov (registration: NCT05832645), and conducted by Biofortis Clinical Research in accordance with the Declaration of Helsinki. All participants provided electronic consent before participating in the study. Data were anonymized and then analyzed. Email and Apple IDs were collected from the participants assigned to the GTM group for user authentication of the prototype app and stored with limited access. Other personally identifiable information was kept in strict confidence. Participants received a US $250 Visa gift card for completing all the surveys given below and were informed that the number and duration of the meditation sessions would not affect the stipend.

### Trial Design

**Figure 1:**
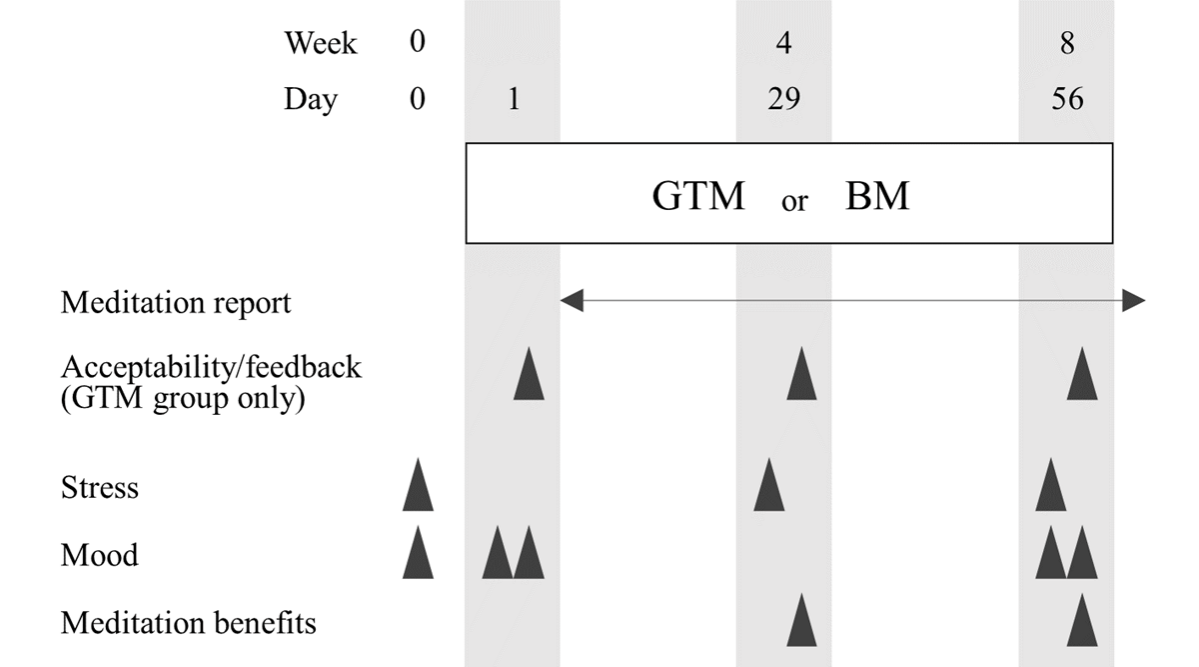
Study scheme. BM: breathing meditation; GTM: guided tea meditation.

A comparator group was established in which participants performed traditional BM without an app or audio guide (active control), and this study used a randomized, unblinded, parallel-group design. Web-based screening was conducted to assess eligibility and perceived stress levels. Given the results, eligible participants were asked to download the Castor Connect mobile app (Castor Connect) and allocated 1:1 to the GTM or BM group. Participants were encouraged to perform their assigned study intervention daily during the 56-day (8-week) intervention period. The duration was set according to the MBSR program. The meditation session performed on the previous day was self-reported by the participants via the mobile app. The acceptability of the prototypes was assessed and participant feedback was obtained only in the GTM group on days 1 (after the first use), 29, and 56 (after the last use). Perceived stress levels were assessed at weeks 0 (screening), 4 (day 29), and 8 (day 56). The mood was assessed before and after the first (day 1) and last (day 56) meditation sessions. A survey to explore the benefits of meditation was conducted at weeks 4 (day 29) and 8 (day 56). All questionnaires were presented and completed via the app. The study scheme and evaluation points are shown in Figure 1.

### Participants

The participants were recruited in May 2023 via email, websites, flyers, and Facebook. The main inclusion criteria were (1) aged 20-49 years; (2) born in the United States; (3) self-reported history of meditation, but no meditation within 30 days (one month) of screening; (4) access to an Apple ID, an email address linked to the Apple ID, and a dedicated smartphone (iPhone 8 or newer models with iOS 16 or newer versions) capable of downloading and running the app; (5) the ability to download, install, and update apps using a smartphone; (6) access to hot water to prepare the matcha green tea beverage; and (7) a willingness to maintain habitual diet (including supplements), lifestyle, and physical activity during the study. The third inclusion criterion was based on the product marketing strategy. The main exclusion criteria were as follows: (1) visual or hearing impairments that could not be corrected with glasses, contact lenses, or hearing aids; (2) self-reported taste or smell impairments within 30 days of screening; (3) a condition that prevented caffeine consumption (eg, caffeine sensitivity and underlying heart condition); and (4) contraindication, allergy, or sensitivity to any components in the study product or allergens present in the facility used to manufacture or pack the study product. A full list of the inclusion and exclusion criteria is available [21]. Based on their responses to the prescreening questionnaires, qualified participants proceeded to provide electronic informed consent for study participation. Eligible participants had a video call with the clinic staff to review the study instructions and confirm when to start the intervention.

### Interventions for the GTM Group

Participants were instructed to perform GTM using the prototype, ekkomi center me (Otsuka Holdings Co Ltd). ekkomi center me consists of matcha green tea and a mobile app that delivers audio guidance to perform meditation while making and drinking the beverage. Audio guidance consists of three parts: (1) a guide for a relaxing introductory exercise, such as progressive muscle relaxation and guided imagery [22,23] (five variations); (2) a guide for meditation with matcha green tea (GTM); and (3) a guide for refocusing. Users could choose a voice actor out of three options and a background sound out of four options every time they used it. The total duration of all audio clips was designed to be approximately 10 minutes, considering time constraints. The contents of the app and audio clips were developed by a psychiatrist and mindfulness experts, as mentioned in the *Acknowledgments* section. Screenshots of the prototype app and an example of the audio script for GTM are provided in Multimedia Appendices 1 and 2, respectively. The flavor of matcha green tea was adjusted for a balanced taste profile with bitterness, sweetness, and umami, and the tea was attached to a stick so that a user could easily make the tea beverage and pay attention to its complex but pleasant taste. The appearance of the prototype matcha stick is shown in Multimedia Appendix 3. The sticks were individually packed so that the participants could carry them.

Participants assigned to GTM received a packet that included the matcha sticks for 8 weeks and instructions to download the prototype app through TestFlight (attached to Multimedia Appendix 4). The participants were encouraged to perform GTM once a day for 8 weeks and were asked to report the time and frequency of meditation. Participants could choose when and where to meditate freely, and they were not prompted to meditate during the intervention period.

### Interventions for the BM Group (as the Active Control)

Participants assigned to BM received a packet that included instructions to perform BM (Multimedia Appendix 5). They were encouraged to perform BM for at least 10 minutes per day according to the instructions. In traditional mindfulness-based programs such as MBSR, practitioners are encouraged to meditate for more than 45 minutes daily [9]. Therefore, the meditation time was not limited. Participants could choose when and where to meditate freely, and they were not prompted to meditate during the intervention period.

### Outcomes

The total meditation time and frequency during the study period were calculated as the primary outcome measures based on the daily meditation reports of the participants. Frequency was defined as the number of days in which the participants completed at least one meditation session. Weekly meditation time and frequency were calculated and compared between the groups as secondary measures.

Changes in stress levels were assessed as secondary outcomes using the Perceived Stress Scale-10 (PSS-10) [24]. The total score ranges from 0 to 40, with higher scores indicating higher levels of stress. Mood metrics were also assessed as secondary outcomes using the Two-Dimensional Mood Scale [25]. In addition to the original 8 components (ie, calm, energetic, irritated, lethargic, listless, lively, nervous, and relaxed), measures of focus and anxiety were assessed using a 6-point Likert scale ranging from 0=Not at all to 5=Extremely. Changes from pre- to postmeditation scores and changes in premeditation scores over the study period were compared between groups. Other benefits of meditation were explored at weeks 4 and 8 by asking participants whether they noticed any positive changes in feelings of happiness, time management, physical discomfort, quality of life, relationships, sleep, and work performance. The answers were collected on a 5-point scale ranging from 1=Strongly Agree to 5=Strongly Disagree.

### Sample Size

Because this was the first study to assess the feasibility and efficacy of GTM, the anticipated effect size was not available. We referred to two previous studies that evaluated mindfulness apps and recruited approximately 100 participants for two arms [20,26]. Under continuous outcome conditions with 100 participants (50 per arm), there was 80% power to detect an effect size of 0.57 using a 2-sided *t* test at a significance level of .05.

### Randomization and Blinding

Participants were randomly assigned to one of the two intervention groups using a variable block randomization model with block sizes of 4 and 6 following an algorithm that automated randomization assignment in the Castor Platform. Randomization was 1:1 stratified by sex (male or female) and the screening score on the PSS-10 (0-17 [below-average stress] and 18-40 [above-average stress]). The average stress levels were based on a mean of approximately 17 in a sample of 2370 American men and women aged ≤45 years [27]. Hence, 100 eligible participants were enrolled in the GTM (n=49) or BM (n=51) group. The study staff and participants were unblinded to the group allocation.

### Statistical Methods

An intention-to-treat (ITT) analysis was performed for all outcomes. The number of minutes of meditation over 56 days was compared between groups using a 2-tailed *t* test. Additionally, the response profile for all continuous outcomes measured over time (eg, stress levels and the weekly number of minutes meditated) was evaluated using a repeated-measures model. The outcome measured at each time point was included in the response vector, including the baseline. The covariance structure for the repeated measures was selected based on the minimization of the corrected Akaike information criteria. The model contained terms for time point, group, and time point by group interaction. Contrast or estimate statements were used to calculate the within-group change from baseline to each specified time point, as well as the between-group difference in the change from baseline. The frequency of meditation was evaluated using a generalized linear model following negative binomial regression and log link. The weekly frequency was explored using a generalized linear mixed model, where the model fit was similar to the repeated-measures model for continuous outcomes measured over time. To analyze within- and between-group changes in mood metrics, the Wilcoxon signed rank test and Wilcoxon rank sum test were used, respectively. Responses to the meditation benefit survey were recategorized (ie, strongly agree or agree, undecided, and disagree or strongly disagree) and compared between the groups using Fisher exact test.

In cases where meditation activity was not reported, the meditation time and frequency for the day were treated as zero. Missing data for stress and mood scores were not imputed, and only observed data were included in the analysis.

All tests were 2-sided and performed at a .05 significance level. Statistical analyses were performed using Statistical Analysis Systems Software (version 9.4; SAS Institute) and R (version 4.2.2; R Foundation for Statistical Computing).

## Results

### Participants

**Figure 2:**
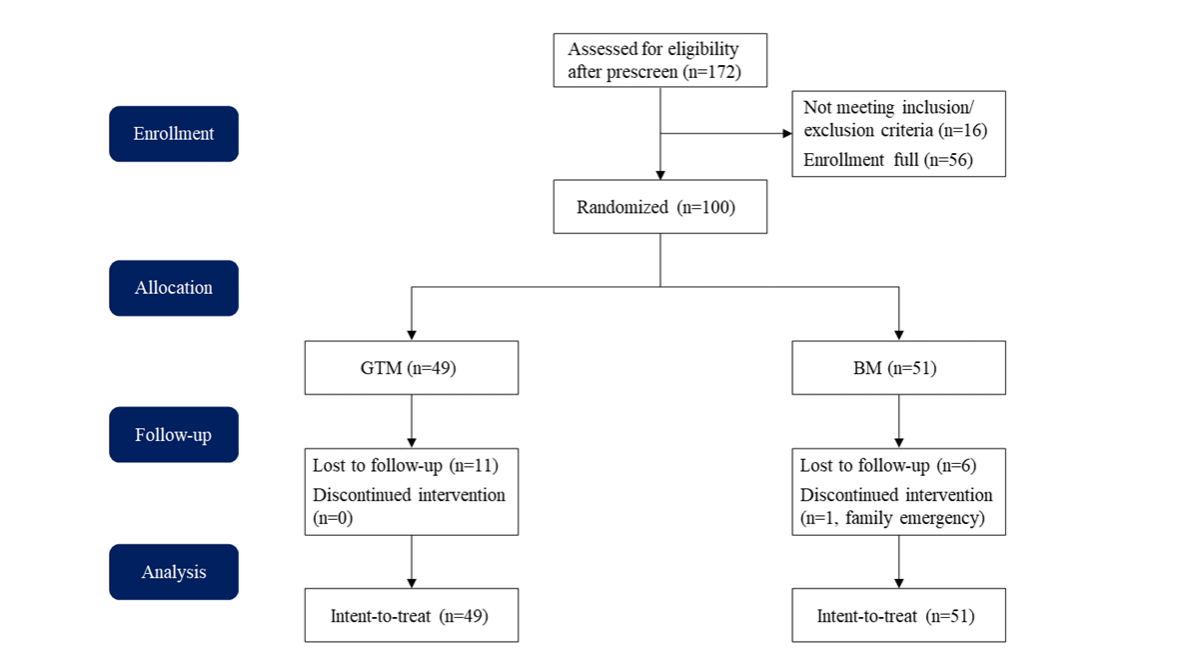
CONSORT (Consolidated Standards of Reporting Trials) participant flow diagram. BM: breathing meditation; GTM: guided tea meditation.

The CONSORT (Consolidated Standards of Reporting Trials) participant flow diagram is shown in Figure 2, and the CONSORT checklist is provided in Multimedia Appendix 6. The recruitment of test participants started in May 2023 and the follow-up ended in July 2023. Regarding retention, 38 (78%) out of 49 participants in the GTM group responded to follow-up surveys at 8 weeks, while 44 (86%) out of 51 participants responded in the BM group.

The participant demographics are presented in Table 1. Of the 100 participants, 81 (81%) participants were female and the average stress level was slightly higher than previously reported values for the US population (~17) [27]. Throughout the study, no adverse events were reported.

**Table 1:** Participant demographics.

Variable		GTM^a^ (n=49)	BM^b^ (n=51)	Overall (n=100)
**Age (years), mean (SD)**		34.5 (7.7)	33.3 (7.9)	33.9 (7.8)
**Sex, n (%)**				
	Female	40 (82)	41 (80)	81 (81)
	Male	9 (18)	10 (20)	19 (19)
**PSS-10^c^ group, n (%)**				
	Below-average stress	15 (31)	16 (31)	31 (31)
	Above-average stress	34 (69)	35 (69)	69 (69)
**PSS-10 screening score, mean (SD)**		19.3 (4.9)	19.9 (5.5)	19.6 (5.2)

^a^GTM: guided tea meditation.

^b^BM: breathing meditation.

^c^PSS-10: Perceived Stress Scale-10.

### Feasibility Assessment

#### Time and Frequency of Meditation (Prototype Use)

The total meditation time and frequency during the study period are presented in Table 2. Neither the time (mean 454.6, SD 219.4 minutes for GTM vs mean 521.0, SD 238.6 minutes for BM; *P*=.15) nor frequency (mean 37.4, SD 17.8 days for GTM vs mean 42.1, SD 16.1 days for BM; *P*=.36) significantly differed between groups.

**Table 2:** Total meditation time and frequency during the 8-week intervention period.

Variable	GTM^a^ (n=49), mean (SD)	BM^b^ (n=51), mean (SD)	Difference, mean (95% CI)^c^	*P* value
Time, total (minutes)	454.6 (219.4)	521.0 (238.6)	–66.5 (–157.5 to 24.6)	.15
Frequency, total (days)	37.4 (17.8)	42.1 (16.1)	0.9 (0.7 to 1.1)	.36

^a^GTM: guided tea meditation.

^b^BM: breathing meditation.

^c^The difference between groups is presented as the model-derived mean and 95% CI.

Weekly meditation time and frequency are listed in Multimedia Appendix 7. Regarding weekly meditation time, the interaction between week and group factors was significant (*P*=.02). There was a tendency that the weekly meditation minutes in the GTM group decreased in the latter half of the 8 weeks, whereas those of the BM group remained constant, and a significant difference was observed between the groups at week 5 (*P*=.04) and 8 (*P*=.045). Regarding frequency, no significant interaction between week and group was detected (*P*=.81), whereas a marginally significant difference was observed between groups at week 5 (*P*=.049).

#### Acceptability of and Feedback on the Prototypes

For the question “Was the app easy to understand and use?” 90% (n=44), 84% (n=41), and 71% (n=35) of the 49 participants answered “strongly agree or agree” on days 1 (after the first use of the prototype), 29, and 56 (after the last use), respectively.

For the question “Do you like the format and content of the audio guidance?” 82% (n=40), 69% (n=34), and 57% (n=28) of the participants answered “strongly agree or agree” on days 1, 29, and 56, respectively. Additionally, 45% (n=22) of the participants provided feedback that they would like more options in the audio clips.

For the question “Did you find the matcha green tea beverage tasty?” 80% (n=39) of the participants answered “strongly agree or agree” on day 1, with 59% (n=29) of them answering the same on days 29 and 56. Of the participants, 37% (n=18) gave feedback that they would like a sweeter taste, and 18% (n=9) commented that they would like more flavor options.

### Efficacy Assessment

#### Perceived Stress Levels

Total PSS-10 scores are presented in Table 3. There was no significant interaction between week and group, indicating no significant difference in the response profiles between the groups (*P*=.29). Within the GTM group, a significant decrease in the PSS-10 score was detected between weeks 0 and 4 (estimate –4.8, 95% CI –6.5 to –3.1; *P*<.001) and between weeks 0 and 8 (–5.5, 95% CI –7.4 to –3.5; *P*<.001). Similarly, within the BM group, a significant difference was detected between weeks 0 and 4 (–4.5, 95% CI –6.1 to –2.9; *P*<.001) and between weeks 0 and 8 (–6.5, 95% CI –8.4 to –4.7; *P*<.001). No significant differences were detected between the groups in the changes from baseline to week 4 (*P*=.79) or week 8 (*P*=.44).

**Table 3:** Changes in perceived stress levels^a^.

Week	GTM^b^ (n=49), mean (SEM)	BM^c^ (n=51), mean (SEM)	Between-group difference, mean (95% CI)^d^	*P* value
0	19.3 (0.7)	19.9 (0.8)	N/A^e^	N/A
4	14.3 (0.9)^f^	15.3 (0.9)^f^	–0.3 (–2.6 to 2.0)	.79
8	13.8 (0.9)^f^	13.5 (1.0)^f^	1.1 (–1.6 to 3.8)	.44

^a^Perceived stress levels were assessed using the Perceived Stress Scale-10.

^b^GTM: guided tea meditation.

^c^BM: breathing meditation.

^d^The difference between groups in the change from week 0 is presented as the model-derived mean and 95% CI.

^e^Not applicable.

^f^*P*<.001 versus week 0.

#### Mood

The pre- and postmeditation mood scores and changes are presented in Table 4 (data at the first meditation session on day 1) and Multimedia Appendix 8 (data at the last meditation session on day 56). During the first meditation session, the within-group change from the pre to postmeditation time point was significant for all items in both groups, except for energetic (GTM, *P*=.34; BM, *P*=.71) and lively (GTM, *P*=.12; BM, *P*=.12). Similarly, during the last meditation session, the within-group changes in both groups were significant, except for energetic (GTM, *P*=.42; BM, *P*=.12), lethargic (GTM, *P*=.95), and lively (GTM, *P*=.98). No significant between-group differences were detected in premeditation or postmeditation scores or changes at the first or last meditation session.

**Figure 3:**
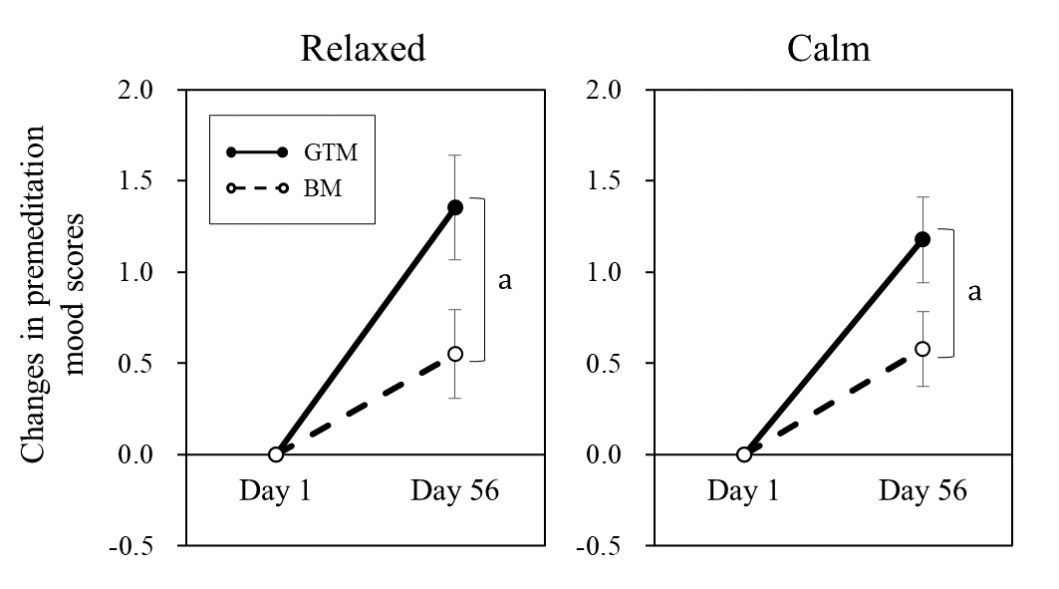
Changes in premeditation relaxed and calm scores from baseline (day 1) to day 56 were significantly greater in the GTM group. Mood was assessed using the Two-Dimensional Mood Scale. Data are presented as mean (SEM). a: *P*<.05. BM: breathing meditation; GTM: guided tea meditation.

Changes in premeditation mood scores from baseline (day 1) to day 56 are presented in Table 5. Significant between-group differences were observed in the relaxed and calm levels (*P*=.04 and .048, respectively; Figure 3).

**Table 4:** Changes in mood before and after the first meditation session on day 1^a^.

Variable	GTM^b^					BM^c^					Between-group *P* value for change
	Pre (n=42), mean (SEM)	Post (n=47), mean (SEM)	*P* value (pre vs post)	Change (post – pre), mean (SEM)	Pre (n=44), mean (SEM)		Post (n=50), mean (SEM)	*P* value (pre vs post)	Change (post – pre), mean (SEM)		
Anxious	1.9 (0.2)	0.9 (0.1)	<.001^d^	–1.0 (0.2)	1.7 (0.2)		0.7 (0.1)	<.001^d^	–1.0 (0.2)	.70	
Calm	2.0 (0.2)	3.3 (0.2)	<.001^d^	1.3 (0.2)	2.2 (0.2)		3.1 (0.1)	<.001^d^	0.9 (0.2)	.15	
Energetic	1.7 (0.2)	2.0 (0.2)	.34	0.2 (0.2)	1.8 (0.2)		1.8 (0.2)	.71	0.1 (0.2)	.66	
Focused	1.8 (0.2)	2.8 (0.2)	<.001^d^	1.0 (0.2)	1.6 (0.2)		2.5 (0.1)	<.001^d^	0.9 (0.2)	.78	
Irritated	1.4 (0.2)	0.4 (0.1)	<.001^d^	–1.0 (0.2)	1.6 (0.2)		0.5 (0.1)	<.001^d^	–1.0 (0.2)	.63	
Lethargic	1.4 (0.2)	0.6 (0.1)	<.001^d^	–0.8 (0.1)	1.5 (0.2)		0.9 (0.2)	<.001^d^	–0.5 (0.1)	.17	
Listless	0.9 (0.2)	0.6 (0.1)	.004^e^	–0.4 (0.1)	1.3 (0.2)		0.7 (0.1)	<.001^d^	–0.6 (0.2)	.25	
Lively	1.5 (0.2)	1.8 (0.2)	.12	0.3 (0.2)	1.3 (0.1)		1.7 (0.2)	.12	0.3 (0.2)	.87	
Nervous	1.4 (0.2)	0.6 (0.1)	.008^e^	–0.8 (0.2)	1.4 (0.2)		0.6 (0.1)	<.001^d^	–0.8 (0.1)	.38	
Relaxed	1.5 (0.2)	3.3 (0.2)	<.001^d^	1.9 (0.2)	1.6 (0.2)		3.1 (0.2)	<.001^d^	1.6 (0.2)	.28	

^a^Mood was assessed using the Two-Dimensional Mood Scale with minor modifications. Changes from pre- to postmeditation were calculated.

^b^GTM: guided tea meditation.

^c^BM: breathing meditation.

^d^*P*<.001.

^e^*P*<.01.

**Table 5:** Changes in premeditation mood scores during the 8-week intervention period^a^.

Variable	GTM^b^ (n=34), mean (SEM)	BM^c^ (n=38), mean (SEM)	*P* value
Anxious	–0.9 (0.2)	–0.2 (0.3)	.10
Calm	1.2 (0.2)	0.6 (0.2)	.048^d^
Energetic	0.5 (0.3)	0.5 (0.2)	.72
Focused	0.7 (0.3)	0.6 (0.2)	.59
Irritated	–0.7 (0.2)	–0.6 (0.2)	.96
Lethargic	–0.3 (0.2)	–0.2 (0.1)	.49
Listless	–0.3 (0.2)	–0.4 (0.2)	.73
Lively	0.6 (0.3)	0.8 (0.2)	≥.99
Nervous	–0.7 (0.2)	–0.3 (0.2)	.10
Relaxed	1.4 (0.3)	0.6 (0.2)	.04^d^

^a^Mood was assessed using the Two-Dimensional Mood Scale with minor modifications. Changes in premeditation scores from baseline (day 1) to day 56 were calculated.

^b^GTM: guided tea meditation.

^c^BM: breathing meditation.

^a^*P*<.05.

#### Other Meditation Benefits

The categorized meditation benefit responses are shown in Multimedia Appendix 9. More than half of the participants in each group experienced positive changes in feelings of happiness (GTM: 32/49, 65%; BM: 34/51, 67%), time management (GTM: 25/49, 51%; BM: 37/51, 73%), quality of life (GTM: 37/49, 76%; BM: 39/51, 76%), relationships (GTM: 28/49, 57%; BM: 34/51, 67%), sleep (GTM: 26/49, 53%; BM: 33/51, 65%), and work performance (GTM: 30/49, 61%; BM: 31/51, 61%) at week 4. Similar results were observed at week 8. However, the groups displayed no significant differences in the distribution of responses at both time points (all *P*>.05).

## Discussion

### Principal Findings and Comparison With Prior Work

Although the performance of GTM was restricted to once (approximately 10 minutes long) a day, whereas the duration of BM was unrestricted, we expected that the meditation time and frequency in the GTM group would be equal to or higher than those in the BM group. As a result, the total meditation time and frequency did not significantly differ between the groups (Table 2), and weekly meditation time and frequency were even lower in the GTM group at weeks 5 and 8 (Table 3). However, the weekly time and frequency of the GTM group remained above 50 minutes per week and 4 days per week, respectively, throughout the study. Notably, these values, especially the frequency, are not lower than those reported for popular mindfulness apps on the market, although direct comparisons are not feasible due to differing test conditions between the studies [26,28-31]. On the other hand, the weekly meditation time in the BM group remained constant at approximately 65 minutes throughout the 8 weeks, although existing research consistently reports a decrease in engagement with mindfulness over time [32-35]. Moreover, the frequency in the BM group ranged from 5 to 6 times a week, indicating that the participants succeeded in performing BM almost every day as instructed. In general, meditating on a daily basis is considered difficult for most people due to several barriers [10,36]. However, it seemed that the population of this study, who had experience with meditation and discontinued it before study participation, did not perceive the daily implementation of BM as a burden. Although the BM group received only brief instructions on how to perform BM (Multimedia Appendix 5), none of the participants required additional instruction or assistance.

It should also be noted that 22% (11/49) and 14% (7/51) of the participants in the GTM and BM groups, respectively, were lost to follow-up or discontinued the intervention during the study. Although these attrition rates were not significantly higher than those of previous studies involving digital mental health interventions [20,36-38], attrition did affect time and frequency outcomes, as we adopted an ITT analysis. Considering participant feedback and decreases in weekly meditation time in the GTM group during the latter half of the 8 weeks, some participants may have become bored with the limited variety in the prototype offerings (5 types of audio scripts and 1 matcha flavor) and stopped using them. Downloading the prototype app through TestFlight (the procedures are shown in Multimedia Appendix 4) might be a hurdle for some participants. In addition, preparing for the environment to listen to audio, the matcha stick, and hot water might have been constraints for the GTM group. However, no obvious feedback was provided regarding this point, and more than half of the users showed acceptance of the prototype app, audio, and matcha throughout the 8 weeks. In conjunction with the meditation engagement data, we believe that GTM exhibits good feasibility. However, further research is needed to expand the variety of offerings.

The effects of GTM on perceived stress levels and mood were investigated and compared with those of BM. The BM intervention that did not involve an app or audio guidance significantly reduced stress levels after 4 weeks. Similarly, GTM showed significant reductions in approximately 5 points of the PSS-10, which are similar to, or greater than, values reported by previous mobile health studies [26,28,39-41]. Direct comparisons with other mindfulness apps with audio guidance are desirable for future studies. A single session of BM significantly and consistently improved mood metrics after the first (day 1) and last (day 56) meditation sessions. Similar effects of GTM were observed and were sustained on day 56. Notably, the changes from day 1 to day 56 in the premeditation relaxed and calm scores were significantly greater in the GTM group. The greater changes in relaxed and calm levels observed in the GTM group may be attributed to the L-theanine contained in the matcha green tea. The relaxing effect of L-theanine has been demonstrated [42,43]. In the GTM group, the average premeditation score of relaxed and calm changed over 56 days, from 1.5 (SEM 0.2) to 2.9 (SEM 0.2) and from 2.0 (SEM 0.2) to 3.2 (SEM 0.2), respectively. In other words, the average participant in the prototype group experienced improvements in their relaxed and calm levels by more than one stage (eg, “somewhat” relaxed to “moderately” relaxed) in the state of premeditation. Elevated relaxed and calm levels may generate well-being and equanimity among practitioners, which needs to be investigated in future studies.

The majority of participants in both the BM and GTM groups experienced positive changes in happiness, time management, quality of life, relationships, sleep, and work performance as they continued the assigned meditation. However, these results should be interpreted with caution because the questionnaire used to assess these exploratory outcomes has not been validated. Future studies should conduct specific, validated surveys to assess each outcome.

### Strengths and Limitations

A strength of this study is that a comparator group was set as the active control (traditional BM), whereas several relevant previous studies set a waitlist group as the control [26,38,44]. Another strength is that ITT analysis was applied to all outcomes, showing the stress-reducing and mood-improving effects of GTM. To the best of our knowledge, this is the first study demonstrating such effects of mindful eating–based practices by ITT analysis. Moreover, this study showed improvements in relaxed and calm levels following multiple GTM sessions were greater than those following multiple BM sessions.

This study does have some limitations. First, all data collected relied on a self-report method. Therefore, the reported meditation activity may not fully reflect actual activity, and data on the efficacy of meditation may be affected by expectation or placebo effects. After randomization, participants received information regarding whether their intervention was the intervention of interest (GTM) or the comparator. Second, correction for multiple comparisons was not conducted for mood metrics considering the exploratory nature of the study. To generalize the findings of this study, additional research needs to be conducted with a limited number of outcomes and a larger sample size. Third, matcha green tea consumption was limited to once a day, as this was the first long-term study to allow participants to consume the prototype matcha. Confirming the safety of the 8-week consumption of the matcha, we do not plan to limit the number of matcha consumption or GTM in commercial settings. Future studies should reflect real-world settings and be conducted with a longer evaluation period. Fourth, demographics such as the experience of meditation, the use of mindfulness apps, and socioeconomic status, which may have acted as confounders, were not investigated. Fifth, we did not assess trait mindfulness as our focus was on emotional state. Future studies should use validated questionnaires to assess mindfulness, such as the Five Facet Mindfulness Questionnaire and the Mindfulness Attention and Awareness Scale. Last, although the set of prototypes showed good feasibility and efficacy, we could not determine which parts of the offerings, to what degree, contributed to program adherence and efficacy.

### Conclusions

Given the time and frequency of GTM and the acceptability of prototypes, we believe that GTM exhibits good feasibility. Meanwhile, GTM reduced stress, improved mood, and let the practitioners feel other benefits, similar to BM. Long-term practitioners of GTM may even feel more relaxed and calmer in the state of premeditation than those who practice BM. To further improve the feasibility and consolidate the efficacy of GTM, the offerings should be improved based on user feedback and compared with other mindfulness apps in future studies with a longer evaluation period.

## Appendix

Multimedia Appendix 1 Screen shots of the prototype app.

Multimedia Appendix 2 An example of the audio script for guided tea meditation.

Multimedia Appendix 3 Appearance of the prototype matcha stick.

Multimedia Appendix 4 Download instructions for the prototype app.

Multimedia Appendix 5 Instructions on how to perform breathing meditation.

Multimedia Appendix 6 CONSORT-EHEALTH checklist (V 1.6.1).

Multimedia Appendix 7 Weekly meditation time and frequency.

Multimedia Appendix 8 Changes in mood before and after the last meditation session on day 56.

Multimedia Appendix 9 Categorized responses to the meditation benefit survey.

## Abbreviations

BM: breathing meditation
CONSORT: Consolidated Standards of Reporting Trials
GTM: guided tea meditation
ITT: intention-to-treat
MBSR: mindfulness-based stress reduction
PSS-10: Perceived Stress Scale-10

## Data Availability

The datasets generated and analyzed during this study are available from the corresponding author upon reasonable request.
