# Supplementary figures and images for: Feasibility and Efficacy of a Novel Mindfulness App Used With Matcha Green Tea in Generally Healthy Adults: Randomized Controlled Trial
Source: JMIR Mhealth Uhealth. 2024 Dec 10;12:e63078. doi: 10.2196/63078 (PMC11668982; doi:10.2196/63078)

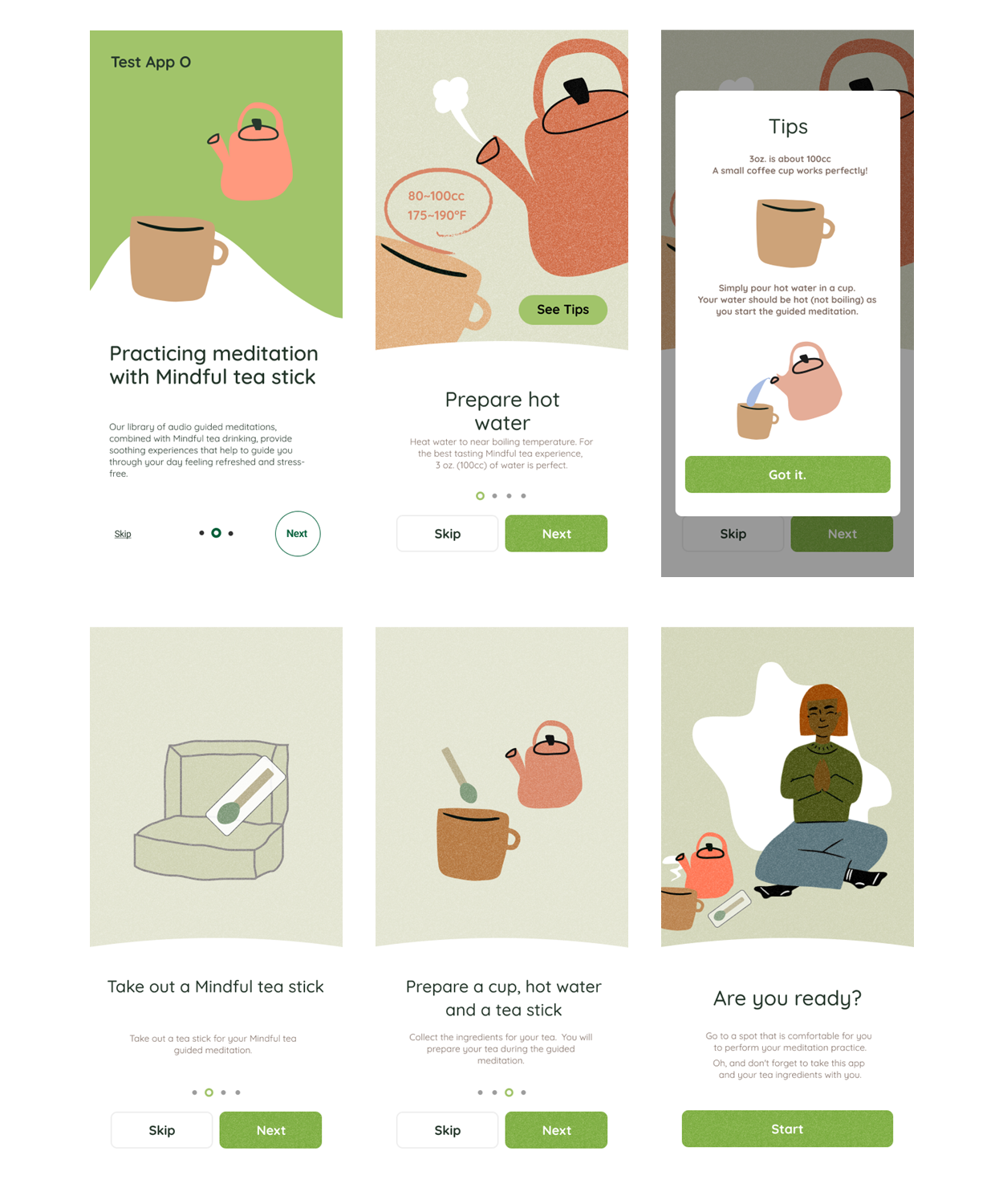

Supplement: Multimedia Appendix 1 [file mhealth_v12i1e63078_app1.png]

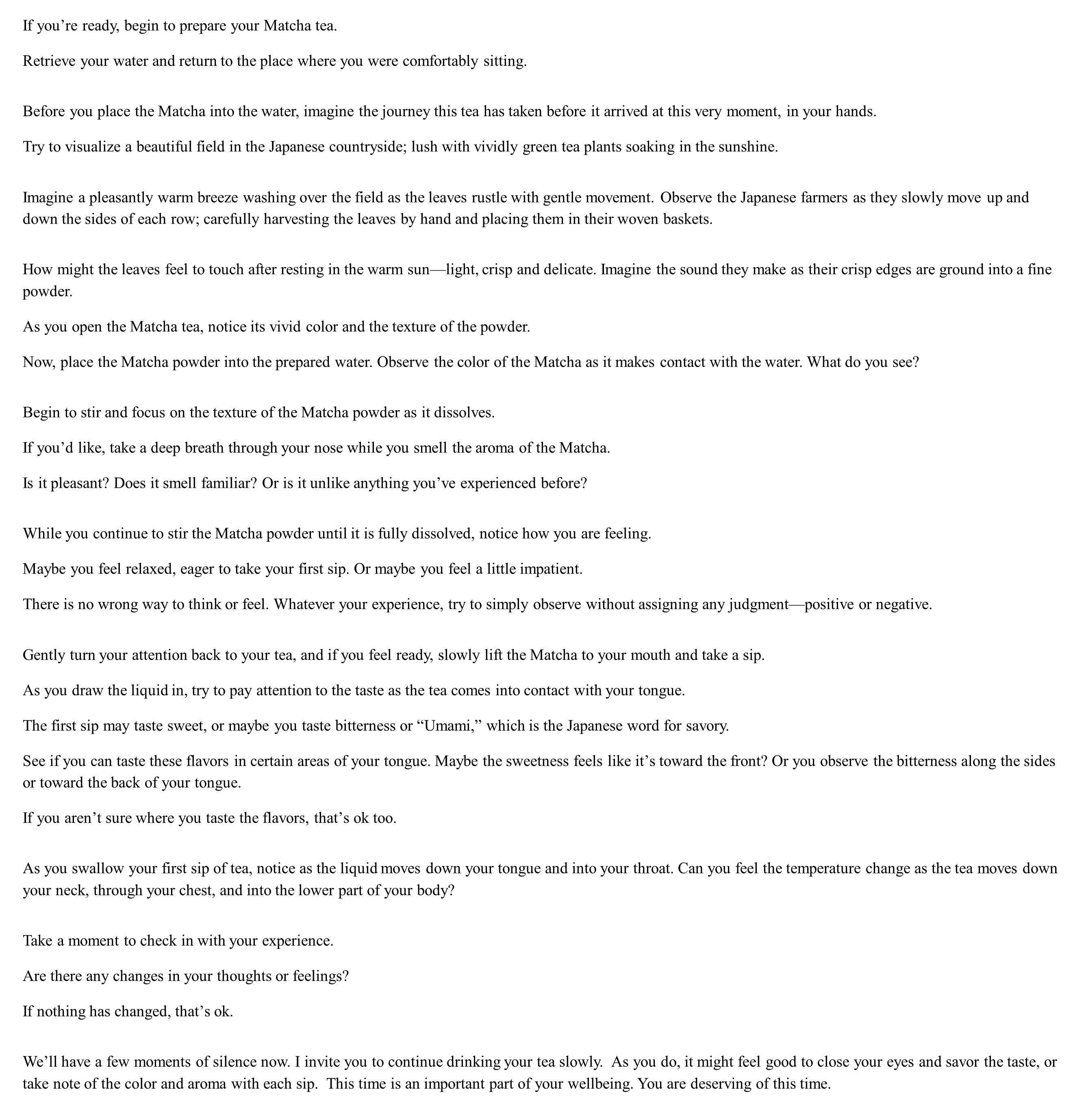

Supplement: Multimedia Appendix 2 [file mhealth_v12i1e63078_app2.png]

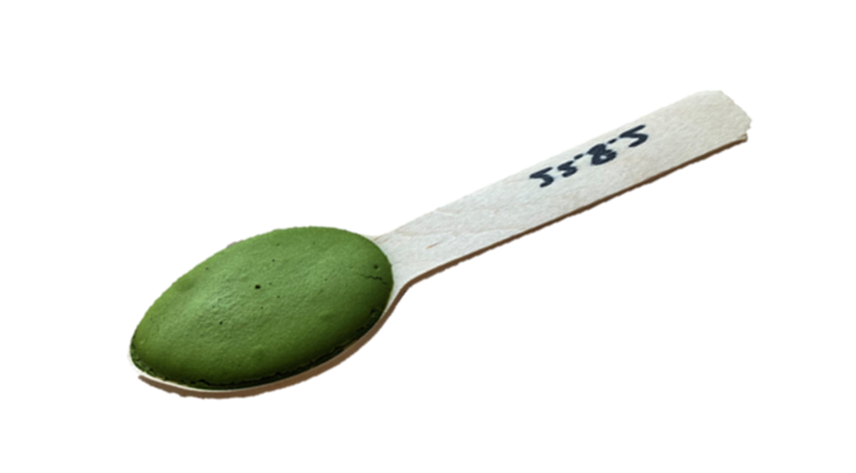

Supplement: Multimedia Appendix 3 [file mhealth_v12i1e63078_app3.png]
