# Supplementary material for: Feasibility and Efficacy of a Novel Mindfulness App Used With Matcha Green Tea in Generally Healthy Adults: Randomized Controlled Trial
Source: JMIR Mhealth Uhealth. 2024 Dec 10;12:e63078. doi: 10.2196/63078 (PMC11668982; doi:10.2196/63078)
Supplement: Multimedia Appendix 4 [file mhealth_v12i1e63078_app4.pptx]

## Slide 1
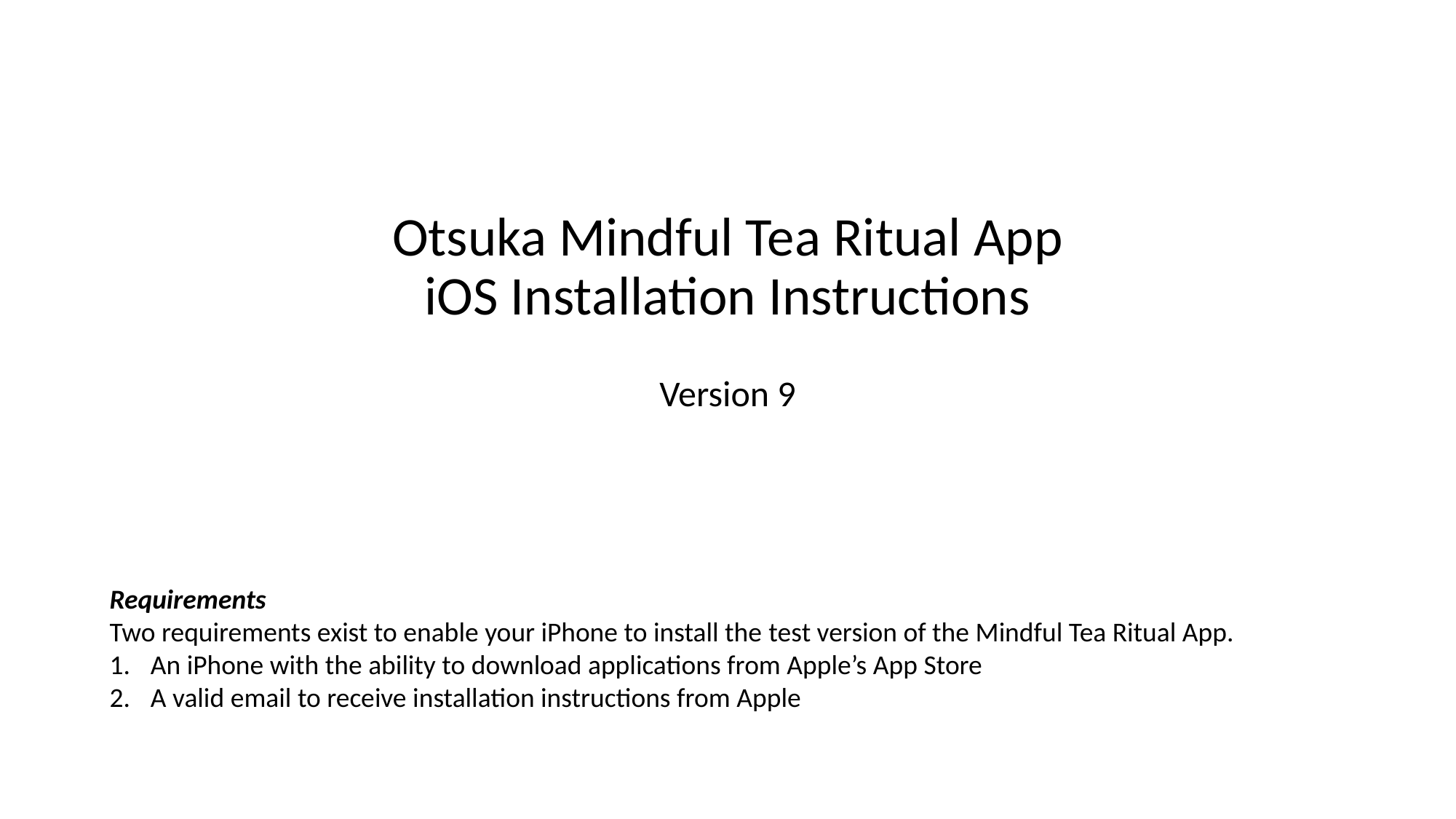

# Otsuka Mindful Tea Ritual App iOS Installation Instructions
Version 9
Requirements
Two requirements exist to enable your iPhone to install the test version of the Mindful Tea Ritual App.
An iPhone with the ability to download applications from Apple’s App Store
A valid email to receive installation instructions from Apple

## Slide 2
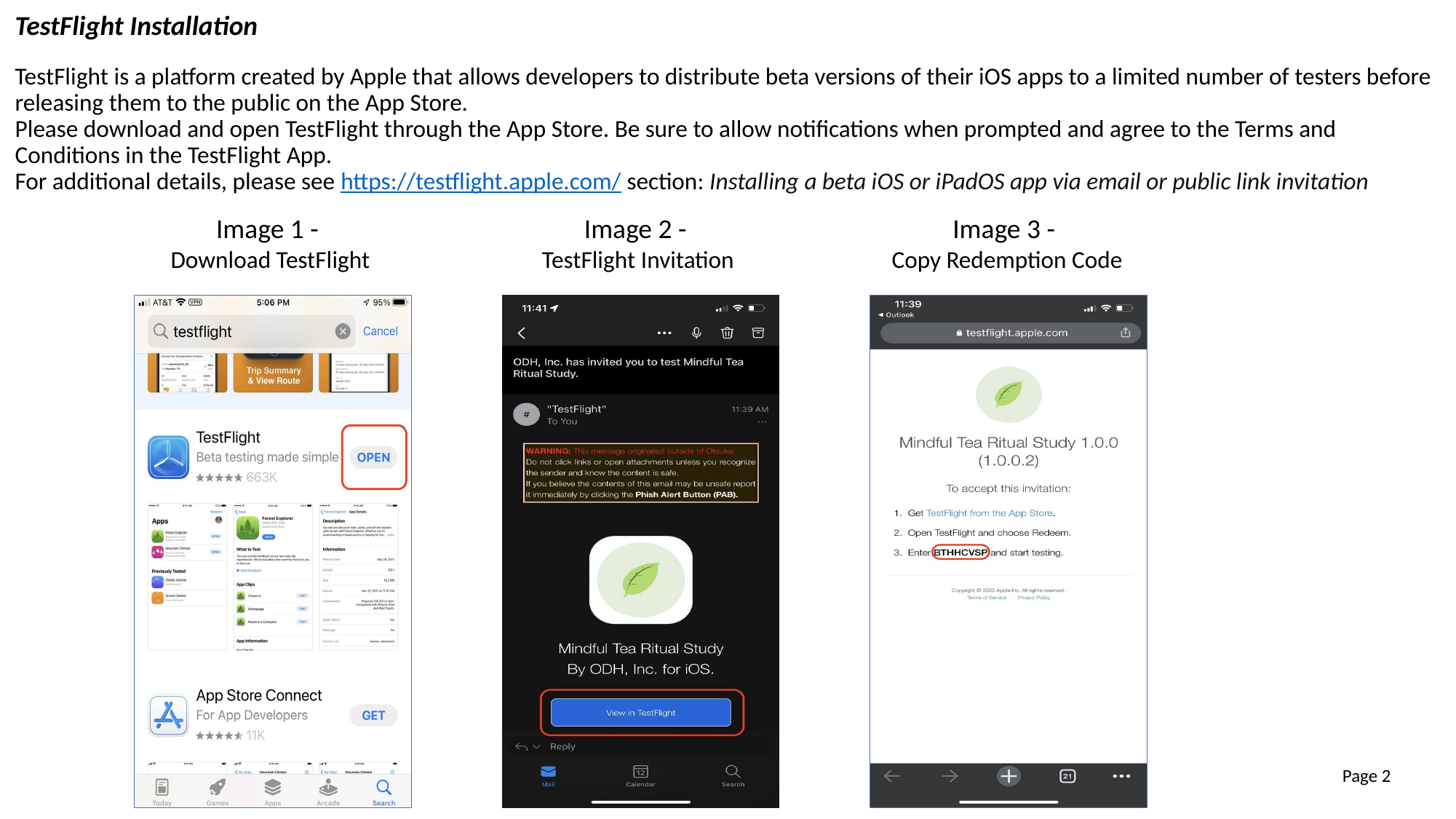

# TestFlight Installation TestFlight is a platform created by Apple that allows developers to distribute beta versions of their iOS apps to a limited number of testers before releasing them to the public on the App Store. Please download and open TestFlight through the App Store. Be sure to allow notifications when prompted and agree to the Terms and Conditions in the TestFlight App. For additional details, please see https://testflight.apple.com/ section: Installing a beta iOS or iPadOS app via email or public link invitation
Image 1 -
Download TestFlight
Image 2 -
TestFlight Invitation
Image 3 -
Copy Redemption Code
Page 2

## Slide 3
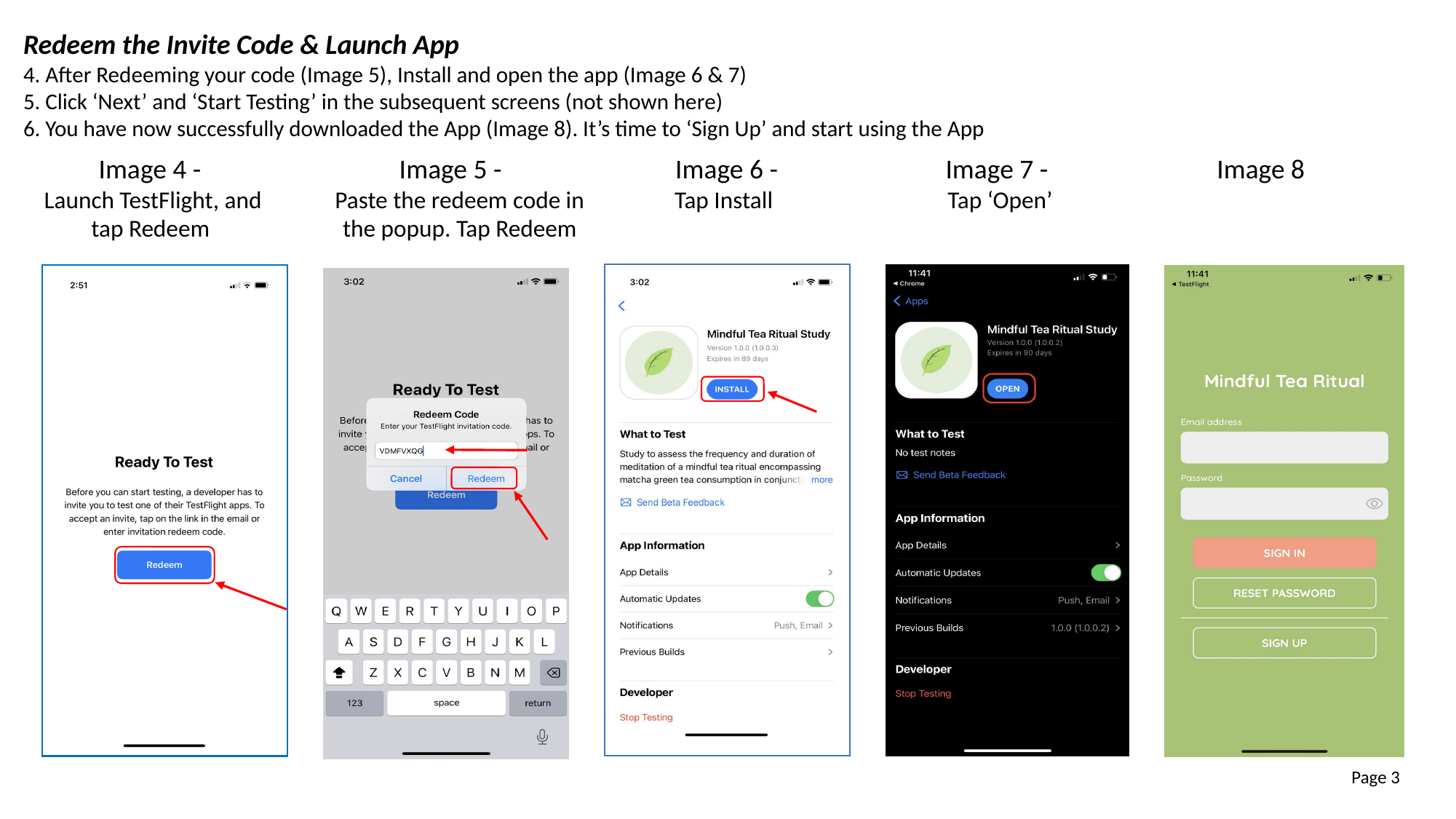

# Redeem the Invite Code & Launch App 4. After Redeeming your code (Image 5), Install and open the app (Image 6 & 7) 5. Click ‘Next’ and ‘Start Testing’ in the subsequent screens (not shown here) 6. You have now successfully downloaded the App (Image 8). It’s time to ‘Sign Up’ and start using the App
Image 4 -
Launch TestFlight, and tap Redeem
Image 5 -   
Paste the redeem code in the popup. Tap Redeem
   Image 6 -   
Tap Install
Image 7 -
Tap ‘Open’
Image 8
Page 3
