# Supplementary material for: Feasibility and Efficacy of a Novel Mindfulness App Used With Matcha Green Tea in Generally Healthy Adults: Randomized Controlled Trial
Source: JMIR Mhealth Uhealth. 2024 Dec 10;12:e63078. doi: 10.2196/63078 (PMC11668982; doi:10.2196/63078)
Supplement: Multimedia Appendix 5 [file mhealth_v12i1e63078_app5.docx]

You are encouraged to perform breathing meditation once a day for 8 weeks. Try to meditate for a minimum of 10 minutes each time. If desired, you can meditate more than 10 minutes per day.

Below is a general guideline on how to perform breathing meditation (adapted from Full catastrophe living, Kabat-Zinn, 1990):

Sit in a comfortable posture that embodies dignity, keeping the spine straight and letting your shoulders drop. Close your eyes and allow your attention to gently align to the sensation of breathing. You can focus on the part of your body where you feel your breath most clearly (for example: nostrils, belly, chest…). Every time you notice that your mind has wandered off your breath, notice what it was that carried you away, and then gently bring your attention back to the sensations associated with your breath.
