# Supplementary material for: Feasibility and Efficacy of a Novel Mindfulness App Used With Matcha Green Tea in Generally Healthy Adults: Randomized Controlled Trial
Source: JMIR Mhealth Uhealth. 2024 Dec 10;12:e63078. doi: 10.2196/63078 (PMC11668982; doi:10.2196/63078)
Supplement: Multimedia Appendix 7 [file mhealth_v12i1e63078_app7.docx]

| Variable and week | | GTM^a^ (n=49),  mean (SD) | BM^b^ (n=51),  mean (SD) | Difference^c^,  95% CI | *P* value |
| --- | --- | --- | --- | --- | --- |
| **Time (minutes)** | | | | | |
|  | 1 | 60.6 (31.6) | 65.1 (26.1) | –4.5 (–16.0 to 6.9) | .43 |
|  | 2 | 64.0 (29.5) | 66.1 (34.2) | –2.1 (–14.8 to 10.6) | .74 |
|  | 3 | 60.1 (29.7) | 58.3 (31.8) | 1.8 (–10.4 to 14.0) | .77 |
|  | 4 | 55.7 (32.2) | 64.9 (36.9) | –9.2 (–23.0 to 4.5) | .19 |
|  | 5 | 55.3 (30.1) | 68.9 (34.4) | –13.6 (–26.4 to –0.7) | .04^d^ |
|  | 6 | 53.9 (33.0) | 66.0 (32.4) | –12.1 (–25.0 to 0.9) | .07 |
|  | 7 | 53.7 (32.9) | 66.0 (35.0) | –12.3 (–25.8 to 1.2) | .07 |
|  | 8 | 51.2 (33.8) | 65.7 (37.5) | –14.5 (–28.7 to –0.3) | .045^d^ |
| **Frequency (days)** | | | | | |
|  | 1 | 4.9 (2.4) | 5.5 (1.7) | 0.9 (0.7 to 1.1) | .16 |
|  | 2 | 5.2 (2.3) | 5.4 (2.2) | 1.0 (0.8 to 1.1) | .62 |
|  | 3 | 5.0 (2.4) | 4.9 (2.3) | 1.0 (0.8 to 1.2) | .94 |
|  | 4 | 4.6 (2.6) | 5.1 (2.3) | 0.9 (0.7 to 1.1) | .30 |
|  | 5 | 4.6 (2.4) | 5.5 (2.2) | 0.8 (0.7 to 1.0) | .049^d^ |
|  | 6 | 4.5 (2.7) | 5.3 (2.3) | 0.8 (0.7 to 1.0) | .09 |
|  | 7 | 4.4 (2.7) | 5.2 (2.3) | 0.9 (0.7 to 1.0) | .12 |
|  | 8 | 4.3 (2.8) | 5.1 (2.4) | 0.8 (0.7 to 1.0) | .06 |

^a^GTM: guided tea meditation.

^b^BM: breathing meditation.

^c^The difference between groups is presented as the model-derived mean and 95% CI.

^d^*P*<.05.
