# Supplementary material for: Feasibility and Efficacy of a Novel Mindfulness App Used With Matcha Green Tea in Generally Healthy Adults: Randomized Controlled Trial
Source: JMIR Mhealth Uhealth. 2024 Dec 10;12:e63078. doi: 10.2196/63078 (PMC11668982; doi:10.2196/63078)
Supplement: Multimedia Appendix 8 [file mhealth_v12i1e63078_app8.docx]

| Variable | GTM | | | | BM | | | | Between-group *P*-value for Change |
| --- | --- | --- | --- | --- | --- | --- | --- | --- | --- |
|  | Pre n=37 | Post n=37 | *P* value (Pre vs Post) | Change (Post – Pre) | Pre n=43 | Post n=43 | *P* value (Pre vs Post) | Change (Post – Pre) |  |
| Anxious | 1.1±0.2 | 0.6±0.1 | <.001^c^ | –0.5±0.1 | 1.3±0.2 | 0.6±0.1 | <.001^c^ | –0.7±0.2 | .48 |
| Calm | 3.2±0.2 | 3.8±0.2 | <.001^c^ | 0.6±0.1 | 3.0±0.2 | 3.7±0.2 | <.001^c^ | 0.8±0.2 | .91 |
| Energetic | 2.4±0.2 | 2.6±0.2 | .42 | 0.2±0.2 | 2.3±0.2 | 2.6±0.2 | .12 | 0.3±0.2 | .85 |
| Focused | 2.5±0.2 | 3.1±0.2 | .002^b^ | 0.5±0.2 | 2.3±0.2 | 3.2±0.2 | <.001^c^ | 0.9±0.2 | .22 |
| Irritated | 0.7±0.2 | 0.3±0.1 | .02^a^ | –0.4±0.1 | 0.9±0.2 | 0.3±0.1 | <.001^c^ | –0.6±0.1 | .42 |
| Lethargic | 1.0±0.2 | 0.9±0.1 | .95 | 0.0±0.1 | 1.2±0.2 | 0.8±0.2 | .02^a^ | –0.5±0.2 | .10 |
| Listless | 0.7±0.2 | 0.5±0.1 | .045^a^ | –0.2±0.1 | 0.7±0.1 | 0.4±0.1 | .010^b^ | –0.3±0.1 | .72 |
| Lively | 2.3±0.2 | 2.3±0.3 | .98 | 0.0±0.2 | 2.1±0.2 | 2.5±0.2 | .012^a^ | 0.4±0.1 | .27 |
| Nervous | 0.7±0.1 | 0.3±0.1 | .008^b^ | –0.4±0.1 | 1.0±0.1 | 0.4±0.1 | <.001^c^ | –0.5±0.1 | .38 |
| Relaxed | 2.9±0.2 | 3.6±0.2 | <.001^c^ | 0.7±0.2 | 2.4±0.2 | 3.5±0.2 | <.001^c^ | 1.1±0.2 | .12 |

Mood was assessed using the Two-Dimensional Mood Scale with minor modifications. Changes from pre- to postmeditation were calculated.

Data are presented as mean ± SEM.

^a^*P*<.05.

^b^*P*<.01.

^c^*P*<.001.
