# Supplementary material for: Feasibility and Efficacy of a Novel Mindfulness App Used With Matcha Green Tea in Generally Healthy Adults: Randomized Controlled Trial
Source: JMIR Mhealth Uhealth. 2024 Dec 10;12:e63078. doi: 10.2196/63078 (PMC11668982; doi:10.2196/63078)
Supplement: Multimedia Appendix 9 [file mhealth_v12i1e63078_app9.docx]

| Week | Question | Response | GTM | BM | *P* value |
| --- | --- | --- | --- | --- | --- |
|  |  |  | n=49 | n=51 |  |
| **4** | **Feel Happier** | Strongly agree/Agree | 32 (65) | 34 (67) | .16 |
|  |  | Undecided | 10 (20) | 8 (16) |  |
|  |  | Disagree/Strongly Disagree | 0 (0) | 4 (8) |  |
|  | **Time Management** | Strongly agree/Agree | 25 (51) | 37 (73) | .08 |
|  |  | Undecided | 12 (24) | 5 (10) |  |
|  |  | Disagree/Strongly Disagree | 5 (10) | 4 (8) |  |
|  | **Physical Discomfort** | Strongly agree/Agree | 16 (33) | 24 (47) | .36 |
|  |  | Undecided | 16 (33) | 12 (24) |  |
|  |  | Disagree/Strongly Disagree | 10 (20) | 10 (20) |  |
|  | **Quality of Life** | Strongly agree/Agree | 37 (76) | 39 (76) | .80 |
|  |  | Undecided | 4 (8) | 4 (8) |  |
|  |  | Disagree/Strongly Disagree | 1 (2) | 3 (6) |  |
|  | **Relationships** | Strongly agree/Agree | 28 (57) | 34 (67) | .68 |
|  |  | Undecided | 11 (22) | 8 (16) |  |
|  |  | Disagree/Strongly Disagree | 3 (6) | 4 (8) |  |
|  | **Sleep** | Strongly agree/Agree | 26 (53) | 33 (65) | .54 |
|  |  | Undecided | 14 (29) | 10 (20) |  |
|  |  | Disagree/Strongly Disagree | 2 (4) | 3 (6) |  |
|  | **Work Performance** | Strongly agree/Agree | 30 (61) | 31 (61) | .94 |
|  |  | Undecided | 9 (18) | 11 (22) |  |
|  |  | Disagree/Strongly Disagree | 3 (6) | 4 (8) |  |

Benefits of meditation were explored on a 5-point scale ranging from 1=Strongly Agree to 5=Strongly Disagree. Responses were re-categorized and compared between groups.

Numbers in parentheses refer to the percentage of the total number.

| Week | Question | Response | GTM | BM | *P* value |
| --- | --- | --- | --- | --- | --- |
|  |  |  | N=49 | N=51 |  |
| **8** | **Feel Happier** | Strongly agree/Agree | 31 (63) | 32 (63) | .64 |
|  |  | Undecided | 4 (8) | 8 (16) |  |
|  |  | Disagree/Strongly Disagree | 2 (4) | 3 (6) |  |
|  | **Time Management** | Strongly agree/Agree | 26 (53) | 29 (57) | .89 |
|  |  | Undecided | 5 (10) | 8 (16) |  |
|  |  | Disagree/Strongly Disagree | 6 (12) | 6 (12) |  |
|  | **Physical Discomfort** | Strongly agree/Agree | 17 (35) | 23 (45) | .36 |
|  |  | Undecided | 16 (33) | 12 (24) |  |
|  |  | Disagree/Strongly Disagree | 4 (8) | 8 (16) |  |
|  | **Quality of Life** | Strongly agree/Agree | 31 (63) | 39 (76) | .68 |
|  |  | Undecided | 3 (6) | 2 (4) |  |
|  |  | Disagree/Strongly Disagree | 3 (6) | 2 (4) |  |
|  | **Relationships** | Strongly agree/Agree | 26 (53) | 32 (63) | .86 |
|  |  | Undecided | 9 (18) | 8 (16) |  |
|  |  | Disagree/Strongly Disagree | 2 (4) | 3 (6) |  |
|  | **Sleep** | Strongly agree/Agree | 31 (63) | 38 (75) | .62 |
|  |  | Undecided | 4 (8) | 2 (4) |  |
|  |  | Disagree/Strongly Disagree | 2 (4) | 3 (6) |  |
|  | **Work Performance** | Strongly agree/Agree | 28 (57) | 36 (71) | .24 |
|  |  | Undecided | 8 (16) | 4 (8) |  |
|  |  | Disagree/Strongly Disagree | 1 (2) | 3 (6) |  |

Benefits of meditation were explored on a 5-point scale ranging from 1 = “Strongly Agree” to 5 = “Strongly Disagree.” Responses were re-categorized and compared between groups.

Numbers in parentheses refer to the percentage of the total number.
